# 2DEGs at Perovskite Interfaces between KTaO_3_ or KNbO_3_ and Stannates

**DOI:** 10.1371/journal.pone.0091423

**Published:** 2014-03-13

**Authors:** Xiaofeng Fan, Weitao Zheng, Xin Chen, David J. Singh

**Affiliations:** 1 College of Materials Science and Engineering, Jilin University, Changchun, People’s Republic of China; 2 Materials Science and Technology Division, Oak Ridge National Laboratory, Oak Ridge, Tennessee, United States of America; University of Nebraska-Lincoln, United States of America

## Abstract

We report density functional studies of electron rich interfaces between KTaO_3_ or KNbO_3_ and CaSnO_3_ or ZnSnO_3_ and in particular the nature of the interfacial electron gasses that can be formed. We find that depending on the details these may occur on either the transition metal or stannate sides of the interface and in the later case can be shifted away from the interface by ferroelectricity. We also present calculations for bulk KNbO_3_, KTaO_3_, CaSnO_3_, BaSnO_3_ and ZnSnO_3_, showing the different transport and optical properties that may be expected on the two sides of such interfaces. The results suggest that these interfaces may display a wide range of behaviors depending on conditions, and in particular the interplay with ferroelectricity suggests that electrical control of these properties may be possible.

## Introduction

Oxide electronics is an exciting area that is also of great potential technological importance. This arises from the wide range of properties available in oxides, even staying within a given structural family, such as perovskite. These include high conductivity metallic behavior as well as ferroelectricity and ferromagnetism. These functionalities may lead to new devices that are difficult to construct using conventional semiconductors. Key parameters for the conducting channels of electronic devices are the conductivity, the carrier velocity and the mobility. Furthermore, discoveries of new emergent phenomena, particular metallic 2 dimensional electron gasses (2DEGs) at certain oxide interfaces have led to renewed interest. [1] While initially it was thought that the 2DEGs represented a particular manifestation of physics of strongly correlated electrons at the interface between Mott and band-insulators, it is now recognized that it is a much more general phenomena related to carriers being forced into electronic states at the interfaces by electric fields associated with charge imbalance (shown e.g. by experiments on interfaces between LaAlO_3_ and SrTiO_3_) [2].

The main challenge at present is to find materials systems and methods for producing controlled, usually high carrier density 2DEGs, with high mobility and a variety of functional behaviors. To date almost all work has focused on mixed valent transition element based perovskites, mainly titanates. One explanation for the success with titanates is that Ti readily takes valences between Ti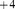 and Ti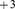 in bulk oxides and that such systems are often metallic as in doped SrTiO_3_, which is a superconductor. [3] The implication is that chemically stable, metallic electron doped titanates can exist. This in contrast to oxides based on metals that do not have mixed valence states as in those cases compensating defects may be expected instead of metallic conductivity. In the perovskite family, besides titanates, both niobates and tantalates have been investigated theoretically, with promising results. [4] Furthermore, electrostatically doped KTaO_3_ at the interface with an ionic liquid has been shown to be superconducting, [5] suggesting that novel physics may also be found at other charged KTaO_3_ interfaces. However, there has been very little work with non-transition metal oxides.

Here we report studies of n-type interfaces based on combinations of K(Ta,Nb)O_3_ and (Ca,Zn)SnO_3_. The motivation is as follows: (1) The oxides on both sides of the interface have mixed valences and can become highly conducting with electron doping and are naturally n-type materials again suggesting the possibility of maintaining electron carriers (i.e. against compensating defects); (2) KNbO_3_ is a well known ferroelectric, where the ferroelectric transition can be tuned down to 0 K by alloying with Ta and similarly ZnSnO_3_ is a ferroelectric [6]–[10] - this provides the possibility of making 2DEGs in proximity to ferroelectric materials and also provides an opportunity for tuning the dielectric constants over a wide range. Note that recent work connects the presence of a high dielectric constant, as is the case in SrTiO_3_, with the occurrence of high mobility due to screening of scattering centers); [11], [12] (3) since both sides of the interface are potentially conducting, it may be that one can arrange the compositions so that one can switch the 2DEG from the Ta/Nb side to the Sn side with e.g. electric field thereby perhaps drastically changing functional properties; and finally (4) it is of interest to explore what properties may be expected for a 2DEG based on a 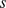 electron system such as ZnSnO_3_ as opposed to the 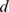-electron systems studied so far. In particular, 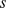 electron materials tend to have much wider bands with potential implications both for the mobility and achievable conductivities.

We note that there are a number of highly conducting but transparent oxide materials, such as Sn doped In_2_O_3_ (ITO) and the recently discovered doped BaSnO_3_. [13]–[18] The high conductivity of doped 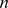-type BaSnO_3_ is indicative of a potential for highly metallic 2DEGs at interfaces of Sn^4+^ perovskites. We note that 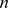-type BaSnO_3_ was prepared early on with doping by Sb and that its band structure was studied emphasizing the 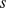-electron character of the conduction band [19], [20].

The lattice parameter of cubic BaSnO_3_, 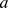 = 4.116 Å, [21]–[23] is larger than most available transition metal perovskites that could be used as substrates for growth. The ∼3% compressive strain that would be imposed in growth of BaSnO_3_ on e.g. KTaO_3_ substrates is within the range that is possible for perovskite growth; in fact epitaxial growth on SrTiO_3_ has been reported. [16], [24] This includes very high mobility (up to 70 cm^2^V^-1^s^-1^) material. [16] Here we focus on CaSnO_3_ and ZnSnO_3_, which have lattice parameters that are more compatible with perovskite KNbO_3_ and KTaO_3_.

CaSnO_3_ occurs in an orthorhombic GdFeO_3_ perovskite type structure characteristic of low tolerance factor materials, [21] while, as mentioned, the related compound ZnSnO_3_ is a LiNbO_3_ type ferroelectric. The LiNbO_3_ structure can be viewed as a highly distorted perovskite structure where the octahedra tilt until blocked by ionic repulsions among the 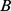-site (Nb) and O ions. This still leaves the 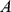-site ions in sites that are too large based on their ionic radii after the tilt. These 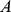-site ions then off-center to obtain a more suitable coordination.

While initially found as a phase formed by ion exchange [6] or high pressure, [7] ZnSnO_3_ can be readily grown by hydrothermal and carbon evaporation methods, [25]–[29] and importantly as heteroepitaxial films on perovskite substrates. [9] KTaO_3_ is a cubic perovskite that is close to ferroelectricity but is not ferroelectric. This absence of a ferroelectric state down to the lowest temperatures is the case in experiment and also in (zero temperature) density functional calculations. [30]–[32] The lattice parameter of KTaO_3_ is 3.99 Å. This is a reasonable match for CaSnO_3_, which has orthorhombic lattice parameters of 5.681 Å, 7.906 Å and 5.532 Å (i.e. 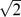
×4.017 Å, 2×3.953 Å and 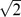
×3.912 Å). KTaO_3_ is an indirect band gap material with an experimental gap of ∼3.6 eV [30], [33].

KNbO_3_ has a smaller gap of ∼3 eV. [34] This difference implies a lower position for the transition metal 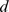 bands, and greater covalency, which has been related to the different ferroelectric properties of these two materials. [32] BaSnO_3_, which is reported to have a cubic perovskite structure at ambient temperature with lattice parameter 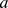 = 4.116 Å and as mentioned is be an excellent n-type transparent conductor with doping.

## Results

### Bulk Compounds

We start with the electronic structure of the bulk compounds. These were based on the experimental cubic structures for BaSnO_3_ and KTaO_3_, 4.116 Å and 3.99 Å, respectively. For CaSnO_3_ we used the experimental orthorhombic lattice parameters (spacegroup 62, 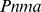, 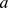 = 5.681 Å, 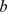 = 7.906 Å, 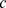 = 5.532 Å), and relaxed the internal atomic positions using the PBE GGA. Similarly, we used the experimental lattice parameters and spacegroup for KNbO_3_ (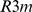, pseudocubic 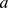 = 4.016 Å) and ZnSnO_3_ (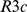, hexagonal setting, 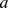 = 5.2622 Å, 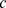 = 14.0026 Å), and relaxed the internal atomic positions consistent with symmetry with the PBE GGA and then calculated electronic structure with the TB-mBJ potential functional.

**Figure 1:**
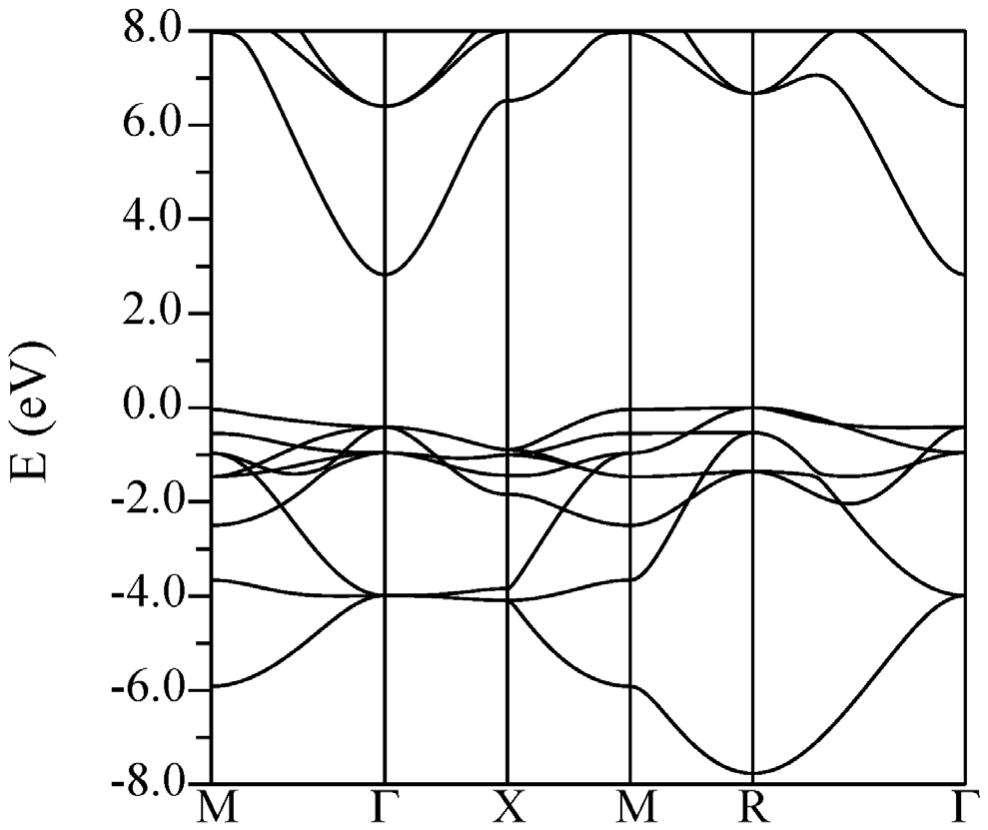
Calculated band structure of cubic BaSnO_3_ using the TB-mBJ potential.

**Figure 2:**
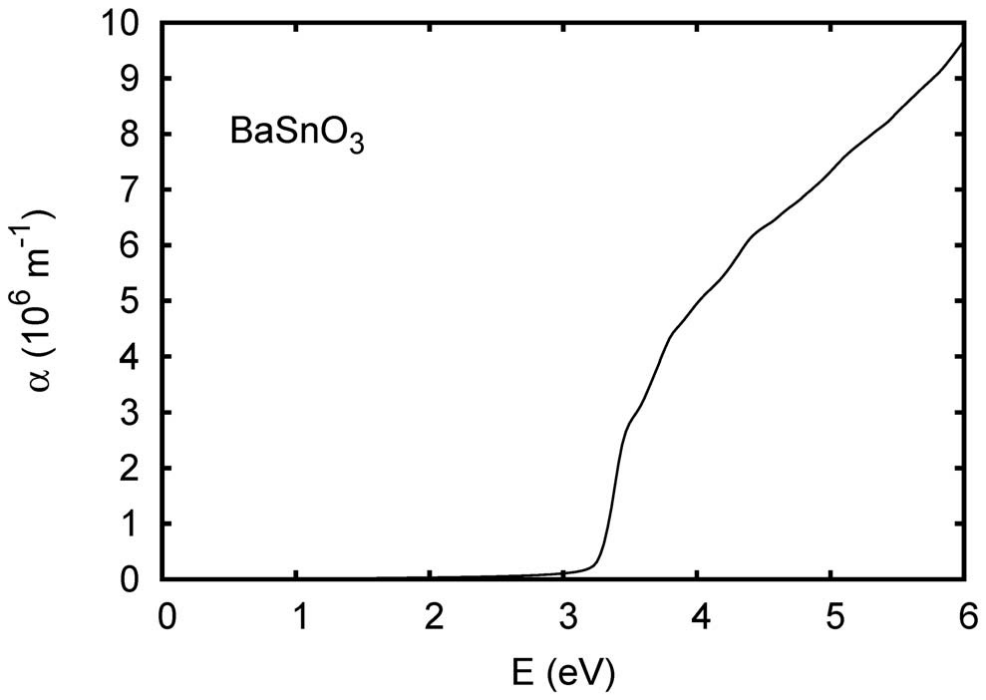
Calculated absorption spectrum of BaSnO_3_ using the TB-mBJ potential. A Lorentzian broadening of 0.025 eV was applied.

The band structure and calculated optical absorption of cubic BaSnO_3_ are shown in Figs. 1 and 2, respectively. The TB-mBJ band structure of BaSnO_3_ is in close agreement with the recent hybrid functional calculations of Liu and co-workers, [35] including the indirect band gap, its value and the structure of the conduction bands. Kim and co-workers observed that while the TB-mBJ functional gives band gaps of III-V semiconductors in good accord with experiment it tends to underestimate band widths relative to hybrid functional and GW calculations. In BaSnO_3_ we obtain a dispersion of the lowest conduction band from Γ-*X* of 3.7 eV as compared to ∼4.3 eV in the hybrid functional calculations of Liu and co-workers, [35] which amounts to a difference of ∼15%. It will be of interest to compare the structure of measured optical spectra with the present calculations (Fig. 2) to determine the band widths. Critical point analysis of ellipsometry data may also be helpful for this.

**Figure 3:**
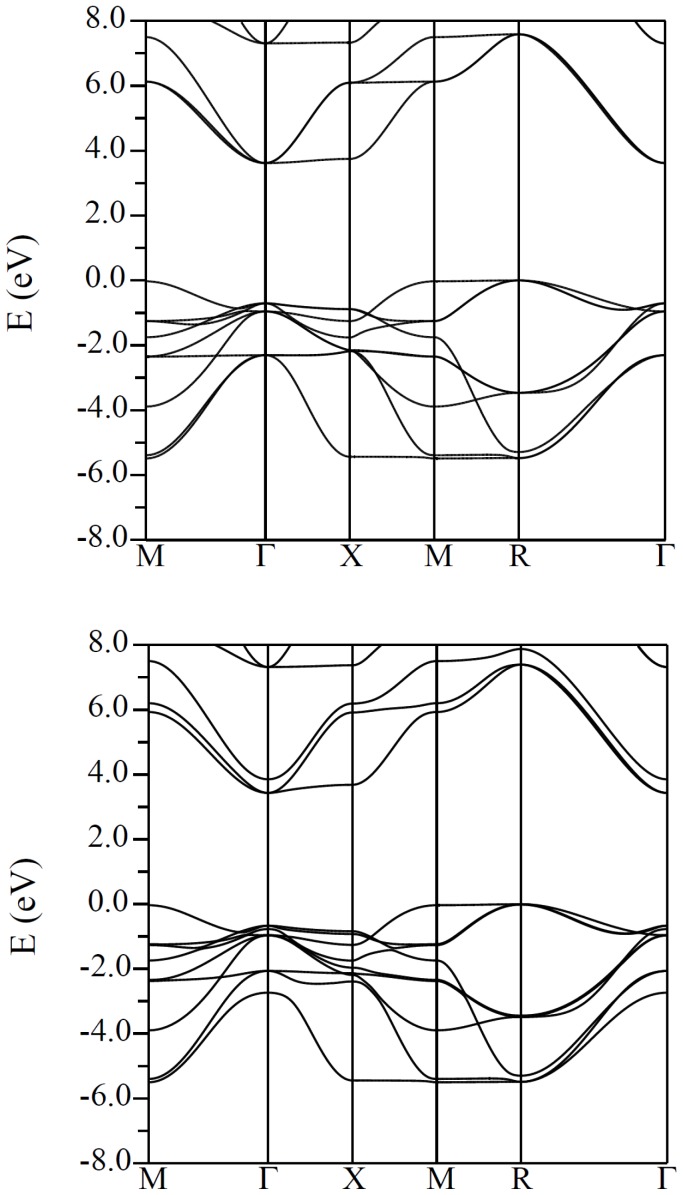
Calculated band structure of cubic KTaO_3_ using the TB-mBJ potential, with a scalar relativistic approximation (top) and including spin orbit (bottom). Note the spin orbit induced splitting of the 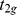 derived conduction band minimum into a two fold degenerate lower lying effective 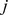 = 3/2 band and a higher lying single degenerate effective 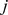 = 1/2 band (considering the 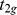 manifold as an effective 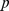 manifold).

As expected, the valence bands have O 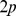 character while the conduction band minimum (CBM) has Sn 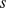 character. The calculated refractive index of BaSnO_3_ is 1.9 at 2 eV. This is approximately 10% smaller than the experimental single crystal value reported by Stanislavchuk and co-workers. [36] The band structure of KTaO_3_ is shown in Fig. 3 The structure of the CBM in the different compounds reflects the different orbital composition. In KTaO_3_ and KNbO_3_ the CBM has transition metal 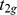 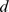 character, i.e. in the absence of spin orbit splitting it is three-fold degenerate at Γ for the cubic compound KTaO_3_ and derives from the 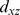, 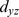 and 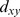 orbitals. These lead to nearly two dimensional bands. This is a consequence of the fact that with the perovskite bonding topology the 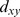 band has much reduced dispersion along 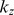 since this dispersion would arise from hopping through the O atom along the 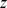 direction, which is not allowed by symmetry, and similarly for the other two 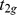 orbitals. This structure is reflected in the band structures of interfacial systems, such as those based on SrTiO_3_ [37].

**Figure 4:**
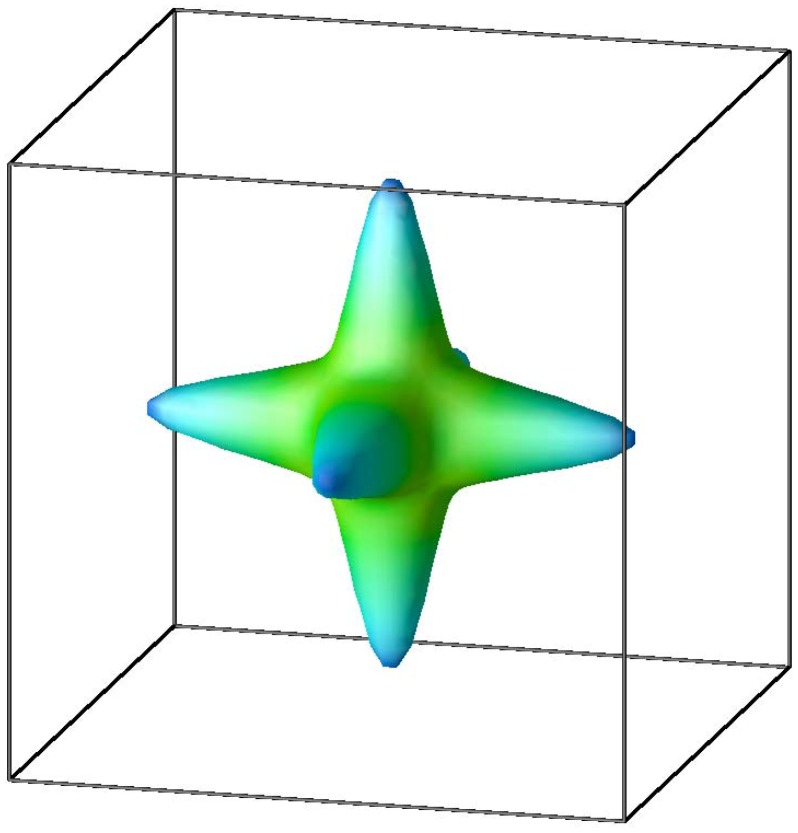
Outermost isoenergy surface for KTaO_3_ above the CBM. There is in addition one inner surfaces (not visible). Note the pronounced non-spherical shape.

In the case of KTaO_3_, the spin-orbit interaction splits these into a lower lying two fold degenerate band that retains this structure and a higher lying single degenerate band that is approximately isotropic. Conventionally, these are described as 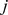 = 3/2 and 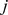 = 1/2 bands, respectively, based on the analogy of the 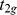 manifold with an effective 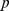 manifold. Fig. 4 illustrates this through an isoenergy surface 0.1 eV above the CBM. In contrast the CBM of the Sn compounds comes from the Sn 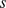 orbital leading to a strongly dispersive isotropic band.

This has consequences for transport. Within Boltzmann transport theory, the conductivity, 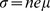 in the degenerate regime at low temperatures is proportional to 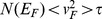, where 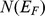 is the density of states at the Fermi energy, 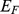, 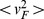 is the average square Fermi velocity in the direction of transport and 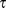 is the effective inverse scattering rate (at finite temperature the formulas are similar, but involve integration with Fermi functions). For an isotropic parabolic band this can be expressed in terms of the effective mass, 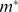, 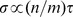. The low temperature (Mott formula) Seebeck coefficient, 
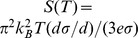
 is proportional to 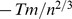 in the single parabolic band case and such expressions involving 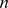 and 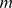 can be obtained for other transport coefficients as well. In deriving these expressions, the effective mass takes two generic roles: (1) in the band velocity, 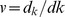 and (2) in the number of carriers, which is given by twice (due to spin) the volume of the Fermi surface, 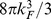, 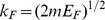, where 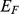 is the Fermi energy. At low 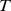, 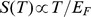. For the anisotropic case (see Refs. [38] and [39] for a discussion of the maximally anisotropic case), where the inverse effective mass is a rank two tensor, 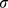 in the light mass direction at a given 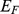 enhanced because the carrier concentration 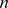 will be higher than for an isotropic system with light mass in all directions, and accordingly the conductivity will be higher if the scattering rate is not proportionately enhanced. One the other hand 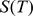 will be the same as in the isotropic light band case, with the same Fermi energy. Conversely, for the same carrier density, 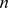, the isotropic light band system will have a higher 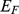 than the anisotropic case and therefore a reduced magnitude of 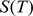. This effect will be particularly pronounced at high carrier densities.

This was discussed previously in the context of the thermoelectric properties of SrTiO_3_/LaAlO_3_ interfaces by Filippetti and co-workers, [40] who pointed out that little is gained in the thermopower from making the electronic structure of SrTiO_3_ two dimensional through the interface, since one already has the effect of a strong anisotropy in the individual bands. It should be noted that the band degeneracy of three also reduces 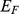 for a given carrier concentration, enhancing 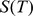 as was noted by Usui and co-workers. [41] We observe that the band structure KTaO_3_ is mostly favorable from a thermoelectric point of view because of the anisotropy (Fig. 4).

**Figure 5:**
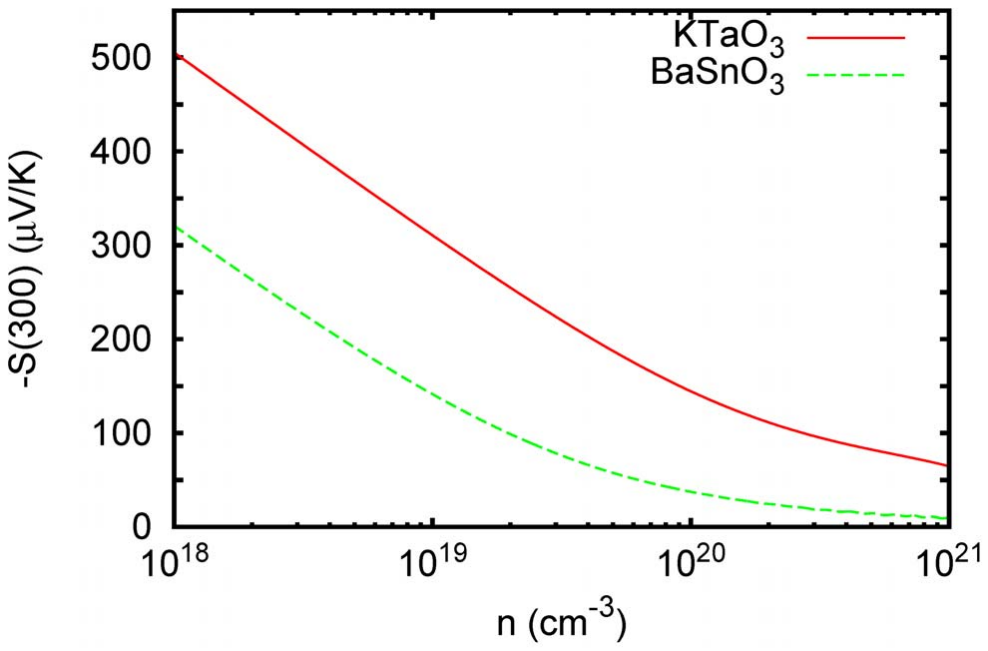
Calculated Seebeck coefficient of n-type KTaO_3_ and BaSnO_3_ as a function of carrier concentration at 300 K as obtained within Boltzmann transport theory with the constant scattering time approximation. The underlying electronic structures are those obtained using the TB-mBJ potential, and in the case of KTaO_3_ includes spin orbit.

BaSnO_3_ is in the opposite limit, with a single Sn 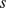 derived conduction band. In this respect it is like ZnO from a transport point of view, and may be expected to show transport behavior consistent with single isotropic parabolic band formulas. Thus, although all materials considered here are perovskite oxides, one can expect very different transport behavior depending on which side of the interface the electron gas resides. On the KNbO_3_/KTaO_3_ side, one expects a lower 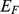 electron gas with degenerate anisotropic bands, while on the ZnSnO_3_/CaSnO_3_ side on expects a high mobility isotropic electron gas with higher 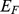 for a given carrier density. To better illustrate the differences in relation to thermoelectricity, we show in Fig. 5 the calculated 300 K thermopower as a function of carrier concentration for BaSnO_3_ in comparison with that of KTaO_3_. This was obtained within Boltzmann theory using the constant scattering time approximation, [42] via the BoltzTraP code, [43] as in our previous work on thermoelectric materials. [44], [45] As may be seen large values are obtained for KTaO_3_, consistent with the earlier work of Usui and co-workers [41] and with experiment, [46] but BaSnO_3_ shows much smaller values at similar carrier concentrations even though the ratio of the effective masses for the conductivity is not so large. As expected, the effect is most prominent at the highest carrier densities.

**Figure 6:**
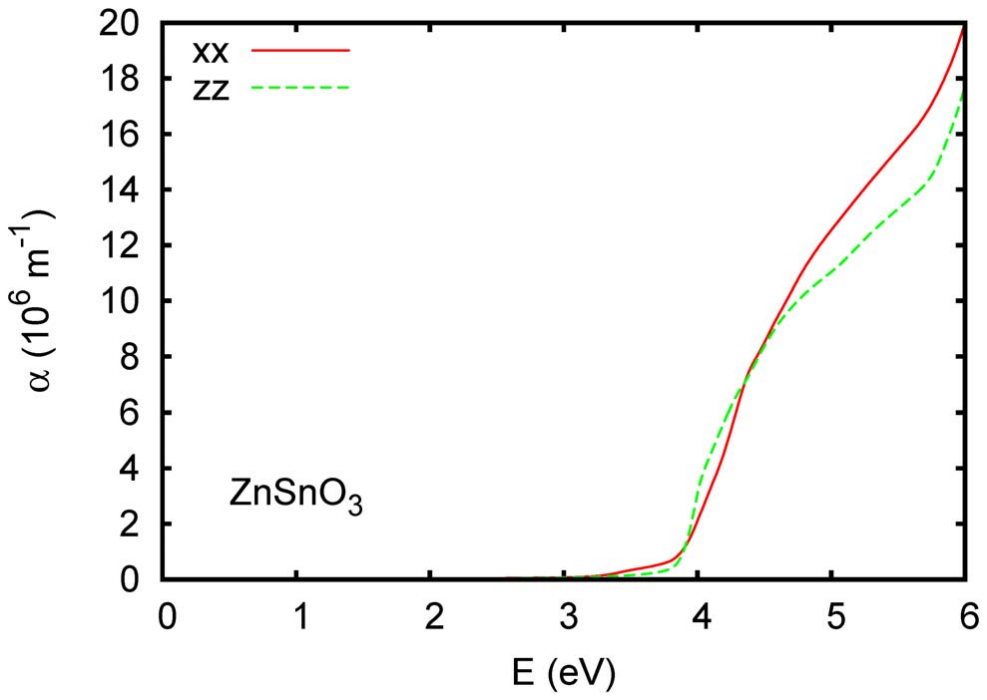
Calculated absorption spectrum of ZnSnO_3_ using the TB-mBJ potential. Here zz is for polarization along the rhombohedral axis and xx is perpendicular. A Lorentzian broadening of 0.025 eV was applied.

**Figure 7:**
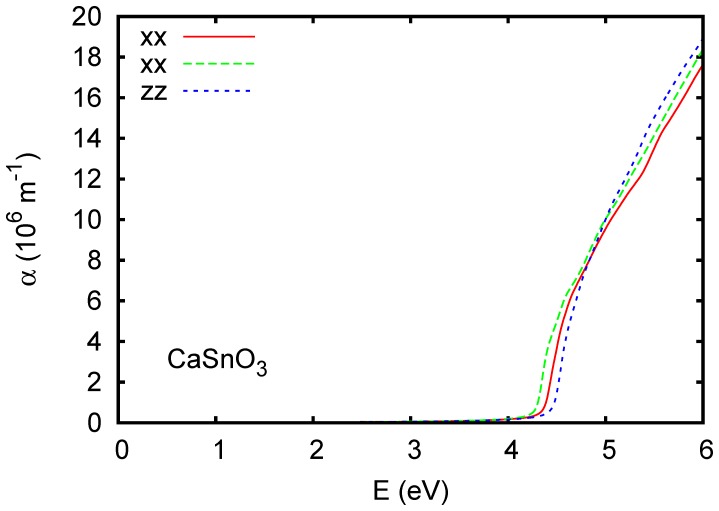
Calculated absorption spectrum of CaSnO_3_ using the TB-mBJ potential. The Cartesian directions are along the crystallographic 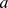, 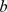 and 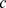 orthorhombic lattice parameters. A Lorentzian broadening of 0.025 eV was applied.

**Figure 8:**
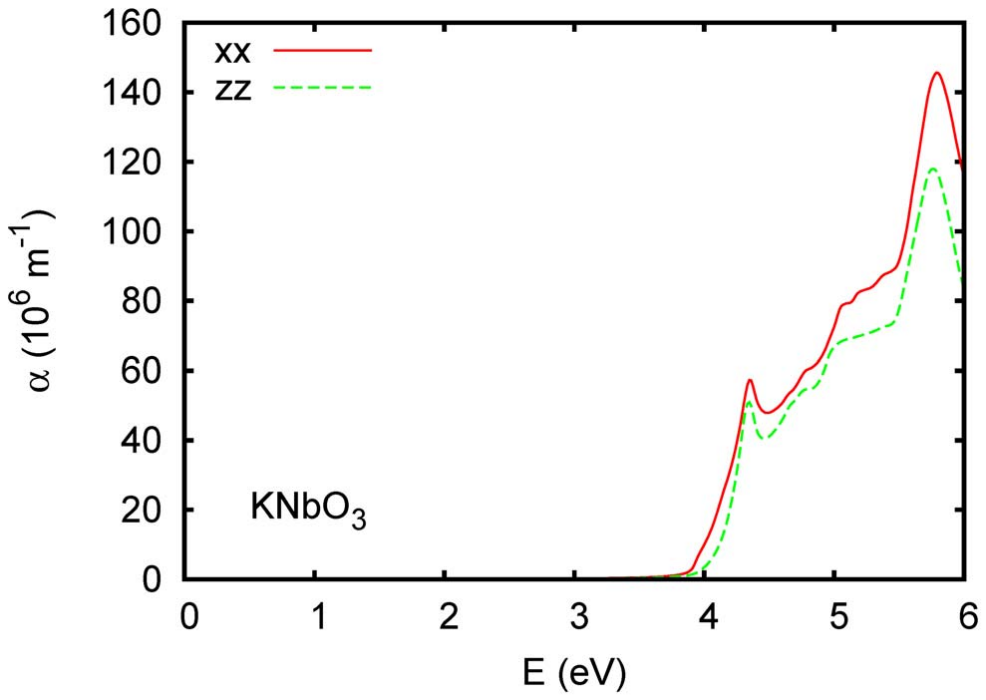
Calculated absorption spectrum of rhombohedral ferroelectric KNbO_3_ using the TB-mBJ potential. Here zz is for polarization along the rhombohedral axis, while xx is perpendicular. Note the different vertical scale from Fig. 2. A Lorentzian broadening of 0.025 eV was applied.

Turning to the optical absorption, it is noteworthy that the magnitude of the absorption in BaSnO_3_ remains rather low at energies above the onset of direct transitions. For example, even at 6 eV (∼3 eV above the onset of direct transitions) 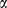 is below 10^7^ m^-1^, in contrast to typical transition metal oxides, where there are strong charge transfer excitations and less dispersive conduction bands. Ferroelectric ZnSnO_3_ (Fig. 6) and orthorhombic CaSnO_3_ (Fig. 7) show stronger absorption above the onset, although still weaker than typical charge transfer gap transition metal oxides. This is illustrated by the calculated absorption spectrum of KNbO_3_, shown in Fig. 8. Wiesendanger reported a strong onset of optical absorption at ∼4 eV in ferroelectric tetragonal KNbO_3_, [47] in qualitative accord with the present results.

We obtained a band gap of 3.43 eV for KTaO_3_, including spin-orbit, in reasonable accord with the experimental value of ∼3.6 eV. This calculation included spin orbit. This significantly affects the band structure due to the high atomic number of the 5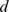 element, Ta (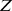 = 73), as has recently been emphasized in the context of 2DEGs at KTaO_3_ interfaces [48].

Without spin orbit (i.e. scalar relativistic) a gap of 3.61 eV is obtained indicating a non-negligible shift of 0.18 eV when spin orbit is included. We also did a calculation for KTaO_3_ based on the self-consistent charge density for a scalar relativistic, including spin orbit for the band structure. This non-self-consistent spin orbit calculation yielded a band gap of 3.45 eV, i.e. practically the same as the self consistent calculation. We include spin orbit in the calculations for the interfaces using this approach, i.e. in the final calculation of the electronic structure based on the charge density from self-consistent scalar relativistic calculations.

**Figure 9:**
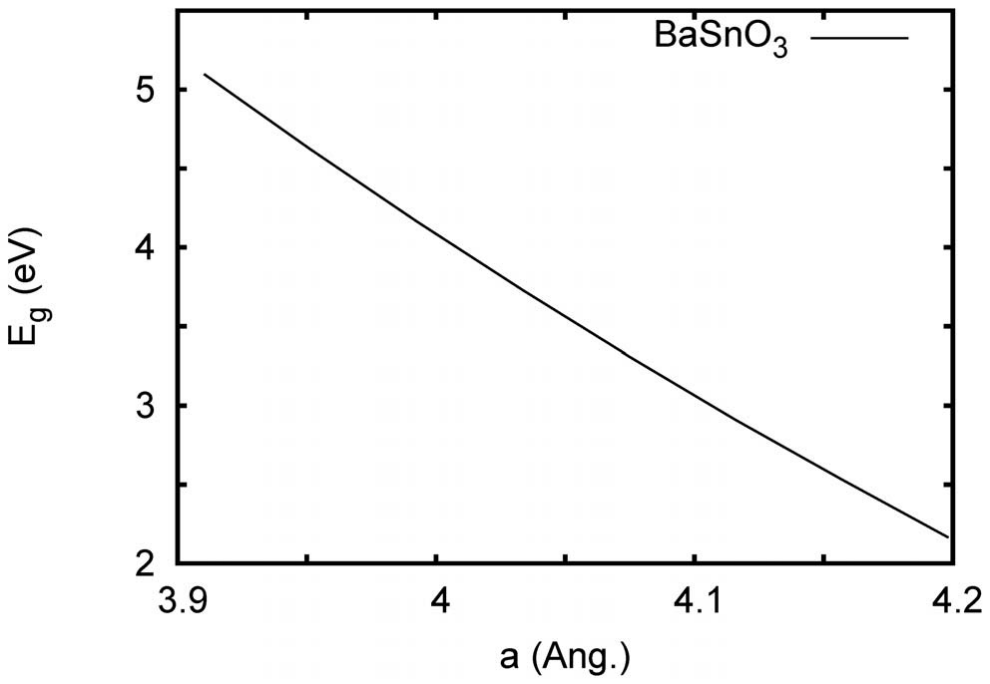
Calculated band gap of BaSnO_3_ as a function of lattice parameter with the TB-mBJ potential.

For BaSnO_3_ we obtain an indirect gap of 2.82 eV, also in accord with experimental reports. For CaSnO_3_, we obtain a substantially larger value of 4.17 eV. This reflects distortion of the very dispersive Sn 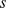 derived conduction bands. The very strong change in the band gap is remarkable and implies strong deformation potentials. It will be of considerable interest therefore to perform low temperature studies of doped n-type perovskite 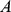
SnO_3_, 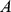 = Ca,Sr,Ba looking for superconductivity. In order to better show the origin of the band gap change with structure we did calculations for BaSnO_3_ both as a function of octahedral rotation and as a function of lattice parameter. For the rotation we considered a frozen in 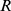-point phonon consisting of alternating (G-type) rotations about a [001] direction. With the experimental lattice parameter we find an instability similar to that reported by Liu and co-workers. [35] We repeated the calculation using the local density approximation (LDA), and also find a weak instability. With the experimental lattice parameter we obtain an energy gain upon 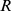-point octahedral rotation about [001] of 5 meV per formula unit with the PBE GGA and 4 meV per formula unit with the LDA. These numbers are comparable to the values obtained for BaZrO_3_ (3 meV per formula unit in the LDA) with the same approach. [49] BaZrO_3_ is a cubic perovskite material that shows no temperature dependent structural transition but for which a small tilt instability exists in density functional calculations. [49]–[51] It will be of interest to measure the low temperature structure of BaSnO_3_ to determine if it is similar to BaZrO_3_ or if there is a distortion in accord with density functional calculations. In any case, we find that the calculated band gap of BaSnO_3_ is only weakly dependent on this distortion. On the other hand we find a strong dependence of the gap on volume. This is shown in Fig. 9, and provides an explanation for the differences between the different compounds.

Turning to the other compounds, for rhombohedral ferroelectric KNbO_3_ we obtain an indirect gap of 2.99 eV, while for ZnSnO_3_ we obtain 3.07 eV. Thus the ordering of the gaps is CaSnO_3_> KTaO_3_> ZnSnO_3_ ≈ KNbO_3_> BaSnO_3_. Considering that the O 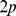 valence bands in perovskites are often aligned, this would suggest that different choices of composition within these systems could yield different results for which side of an electron doped interface has a 2DEG, i.e. a Sn or a Ta/Nb based 2DEG. However, the large difference between the band gaps of BaSnO_3_ and CaSnO_3_ implies a strong sensitivity to structural details, which may play an important role at the interface. Actually, the fact that BaSnO_3_ has the smallest gap would suggest that, all things being equal, the most favorable cases for a 2DEG on the Sn side would be on substrates with the largest lattice parameters, should such substrates become available.

### Supercells

The properties of perovskite thin films are known to be sensitive to strain, which can be controlled in various ways, most commonly choice of substrate, choices of buffer layers and choices of superlattice stackings. Here we are concerned mainly with the question of whether it is possible to have a 2DEG on either side of the interface.

We started with 4 layers of CaSnO_3_ with 6 layers of KTaO_3_. We fixed the in plane perovskite lattice parameter to be 3.99 Å (with a 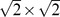 cell), i.e. equal to that of KTaO_3_, and relaxed the 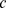-axis lattice parameter, obtaining 39.08 Å, for the 100 atom supercell. This is a condition that would be appropriate for a film of CaSnO_3_ grown on a TaO_2_ terminated KTaO_3_ substrate. We obtained a 2DEG on the Ta side of the interface in this case, as discussed below. Next we replaced KTaO_3_ by KNbO_3_. We kept the same cell parameters as for the KTaO_3_//CaSnO_3_ supercell, but relaxed all internal coordinates. This cell also produced a 2DEG at the electron rich interface on the KNbO_3_ side.

We next replaced Ca by Zn. In this case we investigated a supercell consisting of four layers of KNbO_3_ and six layers of ZnSnO_3_. Also, differently from the CaSnO_3_ case, we relaxed all lattice parameters rather than holding the in-plane lattice parameter fixed. This is a condition appropriate to a thick superlattice. The relaxed lattice parameters were 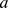 = 5.38 Å, 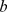 = 5.36 Å, and 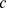 = 41.92 Å. The effective average in-plane perovskite lattice parameter was 3.80 Å, i.e. 4.8% smaller than the CaSnO_3_ based supercells. The resulting 2DEG electron gas is found to be in the ZnSnO_3_ layer, but not at the electron rich interface, but rather at the opposite interface. This is a consequence of the fact that ZnSnO_3_ is ferroelectric (see below).

**Figure 10:**
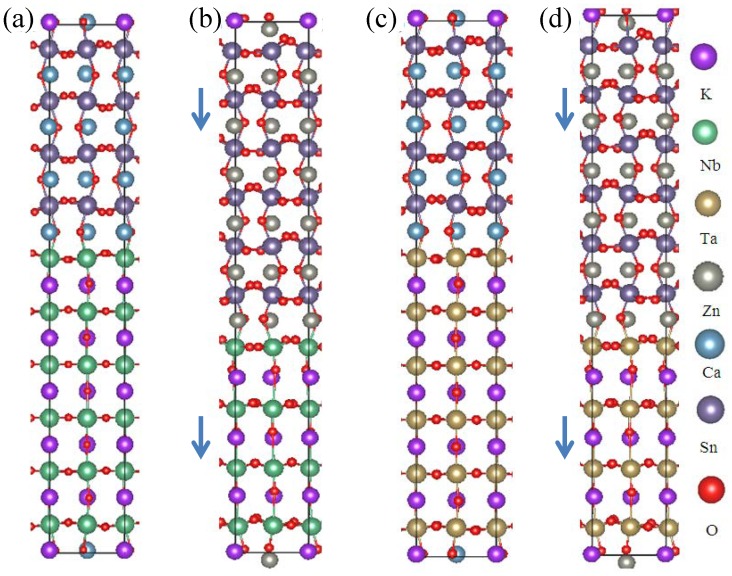
Structures of supercells, left to right (a) KNbO_3_//CaSnO_3_, (b) KNbO_3_//ZnSnO_3_, (c) KTaO_3_//CaSnO_3_, and (d) KTaO_3_//ZnSnO_3_. As seen, the structures containing ZnSnO_3_ have noticeable cation offcentering in the coordinating O cages in both the ZnSnO_3_ and K(Nb,Ta)O_3_ parts of the supercells. This corresponds to a ferroelectric polarization. The direction of this is indicated by the arrows on the left of the corresponding structure figures.

We also did calculations for four layers of KTaO_3_ and six layers of ZnSnO_3_, using the same lattice parameters as the KNbO_3_//ZnSnO_3_ supercell, but again relaxing all internal atomic coordinates. Again in this case, we find the 2DEG in the ZnSnO_3_ at the layer opposite to the electron rich interface. The resulting structures are depicted in Fig. 10. As may be seen, the structures of the CaSnO_3_ and ZnSnO_3_ parts of the supercells are more strongly distorted than the KNbO_3_ and KTaO_3_. The distortions consist of octahedral tilts and 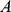-site off-centering, the latter being very pronounced for the ZnSnO_3_ containing supercells.

**Figure 11:**
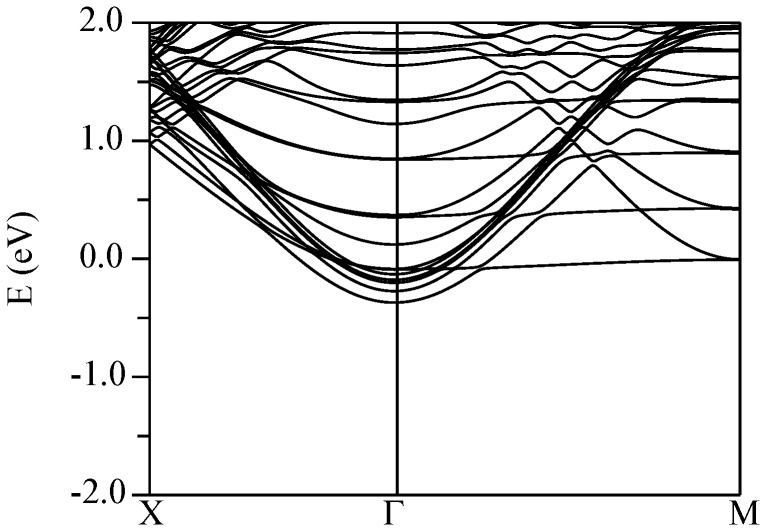
Calculated conduction band structure of the relaxed (KNbO_3_)//(CaSnO_3_) supercell (see text) using the TB-mBJ potential. The Fermi energy is at 0 eV.

**Figure 12:**
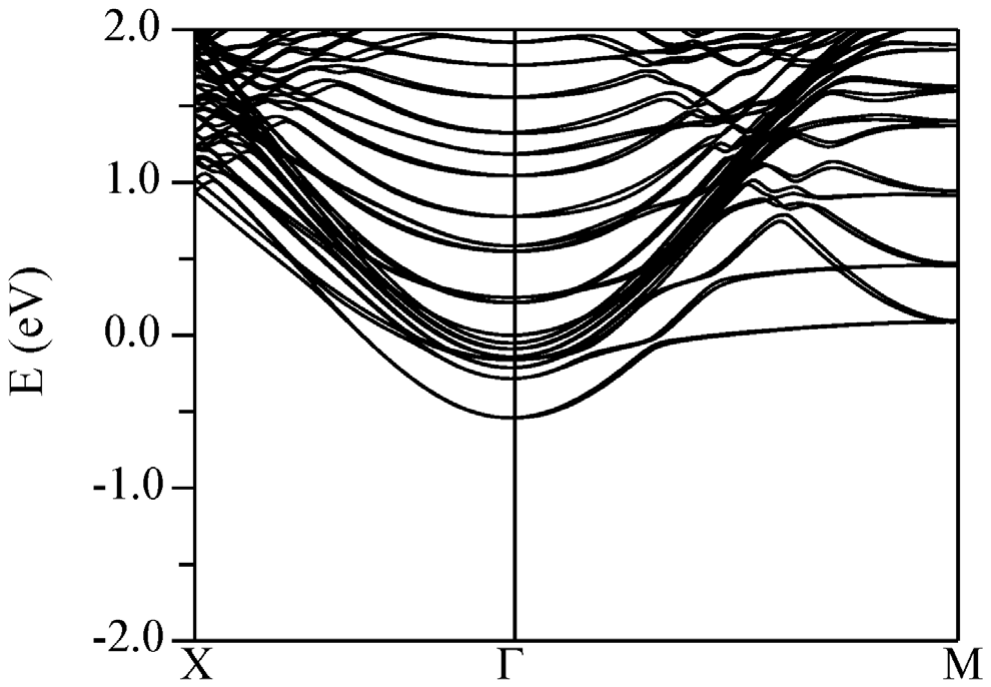
Calculated conduction band structure of the relaxed (KTaO_3_)//(CaSnO_3_) supercell (see text) using the TB-mBJ potential. The Fermi energy is at 0 eV. Spin orbit is included.

**Figure 13:**
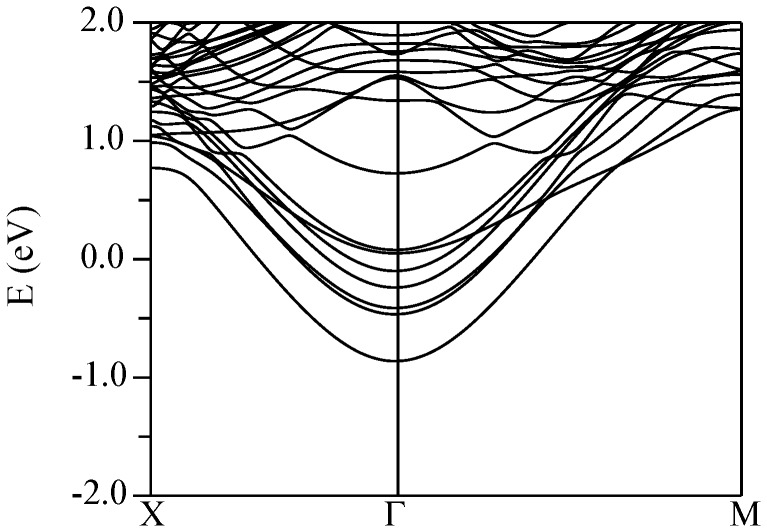
Calculated conduction band structure of the relaxed (KNbO_3_)//(ZnSnO_3_) supercell (see text) using the TB-mBJ potential. The Fermi energy is at 0 eV.

**Figure 14:**
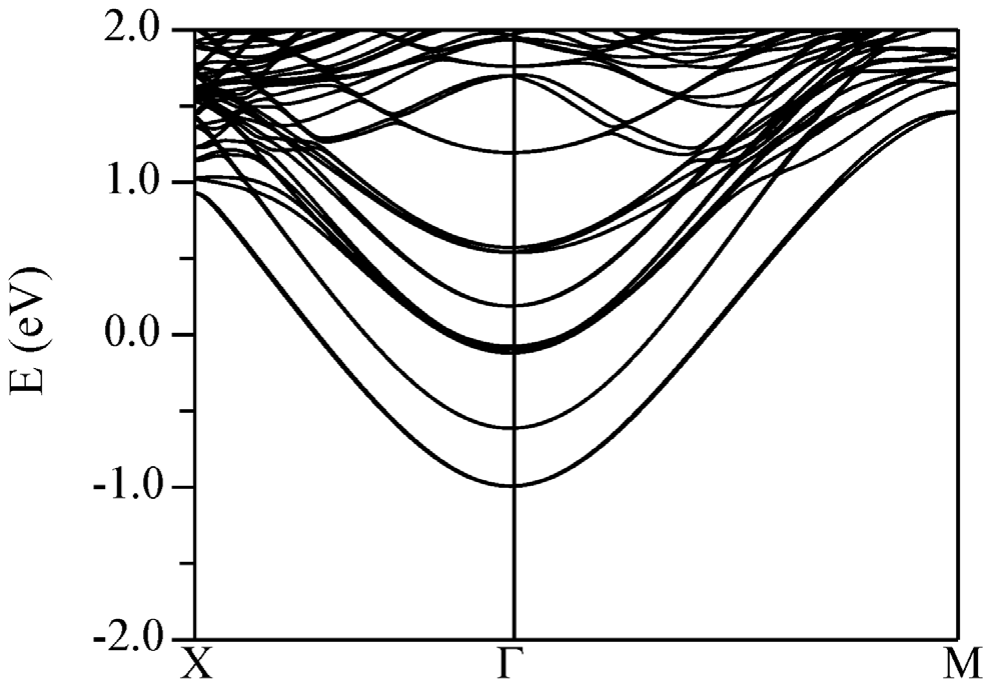
Calculated conduction band structure of the relaxed (KTaO_3_)//(ZnSnO_3_) supercell (see text) using the TB-mBJ potential. The Fermi energy is at 0 eV. Spin orbit is included.

The conduction band structures for the four supercells described above are shown in Fig. 11 (KNbO_3_//CaSnO_3_), Fig. 12 (KTaO_3_//CaSnO_3_), Fig. 13 (KNbO_3_//ZnSnO_3_), and Fig. 14 (KTaO_3_//ZnSnO_3_). In these plots, Γ-*M* is the perovskite [100] direction, while Γ-*X* is the perovskite [110], which is folded at the mid-point due to the 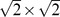 supercell construction. The Fermi energies are fixed by the electron count.

Pairs of bands, in which one member disperses upwards from Γ and the other band is weakly dispersive in the Γ-*M* direction, come from Nb/Ta layers, specifically 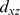 and 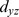 bands in the 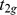 manifold. The 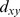 bands, show dispersion along both in plane directions and are split away from the 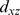 and 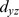 bands. The Sn 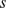 derived bands are also expected to show similar dispersions in both directions. As shown in the band structures, both the CaSnO_3_ based supercells show similar behavior. In particular, the lowest energy conduction bands come from the KTaO_3_ or KNbO_3_ side of the interface and show the structure mentioned above. A series of bands is seen corresponding to the different Nb or Ta layers, but the lowest band is from the layer immediately adjacent to the electron rich interface as might be anticipated. In both supercells the lowest band at Γ is from the 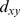 orbital. The next lowest is from the 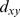 band of the second layer and the 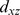/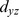 bands of the interface and subsequent layers lie above. The calculated effective masses are 0.45 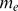 and 0.48 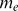 for the KTaO_3_ and KNbO_3_ cases, respectively.

The ordering at the Γ point CBM is a natural ordering for the 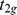 manifold at an interface. This is because hopping is disrupted in the direction perpendicular to the interface, but not in plane. Therefore the width of the 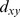 bands is nominally unaffected by the interface, while the 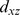 and 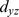 have their width reduced. If the center of the bands is at the same position then the CBM will be formed from the wider band, i.e. the 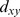 band. The other ingredient that can affect the band ordering is the crystal field.

Crystal field splittings in transition metal oxides come mainly from hybridization between metal 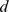 orbitals with the ligand 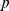 states, in this case, 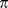 interactions involving the Nb or Ta 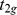 orbitals with the O 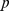 orbitals, so that the conduction bands are nominally 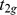 - 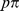 antibonding states. Therefore the 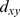 band could be shifted up in energy if the in-plane O atoms were moved closer (or alternatively if the 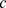-axis O were moved away). However, because the 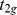 manifold is governed by 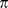 interactions as opposed to the 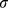 interactions in the 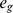 manifold these effects are relatively weaker.

Turning to the ZnSnO_3_ containing supercells, the situation is opposite. We find that the CBM takes Sn 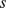 character, i.e. it is on the ZnSnO_3_ side of the interface. As in the case of the CaSnO_3_ supercells, one sees a series of similar bands going up in energy from the CBM, but these are 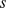 derived bands from different ZnSnO_3_ layers, rather than 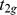 bands. This is readily seen from the near isotropic dispersion away from Γ and is confirmed by projections of the bands (not shown). Importantly, the lowest band, which forms the CBM is not from the Sn 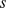 states adjacent the electron rich interface. Instead it is from the opposite, neutral interface. This indicates a ferroelectric tendency in the ZnSnO_3_ similar to that in the bulk. In other words a state with an effective polarization of zero, which would place the 2DEG at the electron rich interface, is less stable than a state with the polarization fixed at the interface planar charge density (50 *μ*C/cm^2^), which then shifts the 2DEG to the opposite interface (note that this is a constrained situation, so it does not make sense to discuss a bulk polarization).

This polarization in the two ZnSnO_3_ containing supercells is seen in the structures (Fig. 10). Specifically the cations on both sides of the interface are visibly displaced from the centers of cages formed by the coordinating O atoms. The direction of this displacement, which is the direction of the ferroelectric polarization is shown by the arrows. As seen, the polarization in the ZnSnO_3_ points away from the charge balanced mixed interface and towards the charge imbalanced interface where the electrons comprising the 2DEG originate, consistent with the above discussion. The polarization in the KNbO_3_ and KTaO_3_ parts of the supercell, which do not contain charge carriers in these cells points in the same direction as that of the ZnSnO_3_.

We note that a related discussion has been presented by Wang, Niranjan and co-workers, who considered 2DEGs at ferroelectric interfaces. [52], [53] They predicted that these may be electrically switchable, with important implications for device applications. Based on our results this could also be the case here.

This may be a particularly interesting form of 2DEG. In particular, because of the ferroelectricity it is shifted away from the charged interface, which may then be viewed as a form of 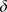-doped 2DEG, i.e. a 2DEG that is in a different physical location than the source of the doping that produces it. It may be then that this could be a particularly high mobility 2DEG if clean, smooth, low defect density capping layers can be grown in practice. The effective mass of the CBM was the same to two figures for both ZnSnO_3_ supercells and was 0.37 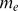, i.e. ∼20% lighter than the KTaO_3_/KNbO_3_ 2DEGs in the CaSnO_3_ based supercells.

The calculations above are for highly idealized systems. In practice, adding 1/2 *e* per unit cell is extreme. As seen in the band structure plots, the Fermi energy is more than 1 eV higher than the CBM for the ZnSnO_3_ cases. While it is known that these Sn compounds can be heavily doped 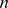-type, in general the chemical stability of a phase decreases as one dopes away from the ideal valence. Specifically, one may expect that the energy of defects that compensate the excess charge will decrease as the Fermi energy is raised and so it may very well be that the realizable carrier density is significantly lower than the idealized cases considered here. Electronic reconstructions have also been discussed in the case of SrTiO_3_/LaAlO_3_, [54] although it should be noted that this is hard to reconcile with the high mobilities observed in this system. Furthermore, it may be expected that the 2DEG will be sensitive to the details of the structure. This is particularly so for the case where one has ZnSnO_3_, as in general ferroelectricity is very sensitive to strain.

However, we expect the qualitative features to remain: (1) depending on details it is possible to have 2DEGs in either the transition metal side or the stannate side of the interfaces; (2) the stannate based 2DEGs have lower effective mass than the transition metal based 2DEGs; and (3) ferroelectricity can shift the 2DEG away from the electron rich interface (presumably this can happen for the ZnSnO_3_, but may also (depending on details such as the strain) occur for strained CaSnO_3_ films or other similar films such as SrSnO_3_ or BaSnO_3_); in this case observation of the 2DEG will depend critically on the termination of the film, e.g. the capping layer. Also, it should be noted that the ordering of the band gaps of the bulk compounds by itself does not predict predict on which side of the interface the 2DEG is found, showing that the details of the interfaces and strain states are important and perhaps can be used to tune these systems.

## Discussion

First principles calculations have been used to study the range of possible behavior for electron rich interfaces between K(Nb,Ta)O_3_ and (Ca,Zn)SnO_3_. We find that depending on details it is possible to produce 2DEGs on either side of the interface. The 2DEGs on the stannate side have lower effective mass. A complicating factor is the interplay with ferroelectricity, which can shift the 2DEG away from the interface when it occurs on the stannate side. This suggests that there may be a very interesting, and perhaps electrically controllable interplay between strain, ferroelectricity and the 2DEG electronic properties in these interfacial systems. This also implies that the possibility of ferroelectricity shifting the 2DEG away from the interface should be kept in mind when interpreting experimental results on interfaces with these stannates, as depending on the conditions it may result in non-observation of an expected 2DEG. This is a general issue for 2DEGs produced by interfaces involving ferroelectric materials. It will be of considerable interest to investigate the behavior of these and related electron rich interfaces between stannates (e.g. ZnSnO_3_, CaSnO_3_, SrSnO_3_ or BaSnO_3_) and transition metal based compounds (e.g. KNbO_3_, KTaO_3_ or even LaTiO_3_) from an experimental point of view.

## Materials and Methods

We investigate the properties of the interfaces using density functional calculations. The results shown are for 100 atom 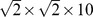 supercells consisting of layers of K(Ta,Nb)O_3_ and (Ca,Zn)SnO_3_ stacked along the [001] direction, with a 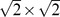 in-plane structure to allow the possibility of rotation/tilts of the octahedra. Similarly, we used even numbers of layers to avoid artificially blocking octahedral tilts that may be important for properly describing the structure. In most calculations we used six layers of K(Ta,Nb)O_3_ and four layers of CaSnO_3_, or four layers of K(Ta,Nb)O_3_ and six layers of ZnSnO_3_. Each case constructed had two interfaces at 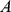
O layers. For one interface we used a mixture of the two cations to construct a charge neutral interface, i.e. KCaO_2_ or KZnO_2_ (balancing the compounds on either side of the layer), while at the other we constructed an electron rich charge imbalanced interface, i.e. Ca_2_O_2_ or Zn_2_O_2_. The excess charge of 1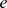 per two perovskite unit cells amounts to 50 *μ*C/cm^2^. This is similar to the polarization of bulk ZnSnO_3_, which has a reported value of 59 *μ*C/cm^2^. [55] This choice of supercell results in non-centrosymmetric cells, which are not usually selected because the absence of inversion symmetry slows density functional calculations. On the other hand, it allows the layers to be truly polar, which turns out to be important in the ZnSnO_3_ containing supercells, as discussed below. As mentioned, we also used a 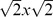 in plane structure to allow octahedral rotations and tilts, i.e. the choices made allow the main classes of perovskite instabilities, arbitrary polar off-centering as well as octahedral tilts.

The atomic positions were determined by structure relaxation using the standard generalized gradient approximation (GGA) of Perdew, Burke and Ernzerhof. [56] No symmetry was imposed in these relaxations. We then calculate the electronic structures using the modified Becke-Johnson potential functional developed by Tran and Blaha, [57] which we denote TB-mBJ. This potential, unlike standard GGA functionals, which are designed to reproduce total energies, gives band gaps in reasonable accord with experiment for many simple semiconductors and insulators [58]–[61].

Initial relaxations were done using the VASP code, [62], [63] while final relaxations of the atomic positions and electronic structures were done using the linearized augmented planewave (LAPW) method [64] as implemented in the WIEN2k code. [65] Well converged basis sets were used. In particular, we used local orbitals with the standard LAPW augmentation to accurately include semicore states (i.e. the LAPW+LO method). [66] The standard LAPW augmentation was used for all other states as well, except that the augmented planewave plus local orbital (APW+lo) basis [67] was used for the O 2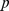 states to accelerate convergence. In all cases we used metal sphere radii substantially larger than for the O. This was done to give good convergence for the metal atoms using the standard LAPW+LO method, which gives an accurate treatment in cases with semicore states as is required for early transition elements such as Nb and Ta as well as for the 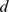 states of Zn (see the discussion in Refs. [66] and [68]). For O better convergence was obtained using the APW+lo method for the 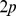 states, since there is not semicore in this case and the small sphere radius makes linearization errors negligibly small. The sphere radii used in the supercells were 2.1 Bohr for K and Ca, 2.25 Bohr for Sn, 1.85 Bohr for Ta, 2.0 Bohr for Nb, 2.05 Bohr for Zn and 1.4 Bohr for O except for the KNbO_3_/CaSnO_3_ supercell for which an O radius of 1.45 Bohr was used.

We used dense Brillouin zone samplings, which were needed especially because of the dispersive nature of the Sn s-derived conduction bands. These amounted to at least 8×8 in-plane meshes for the 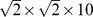 supercells. Optical properties, shown for some of the bulk compounds, were calculated using the electric dipole matrix elements via the optical package of the WIEN2k code. For this we used denser three dimensional k-point grids of 16×16×16 for the cubic compounds and similarly dense grids in the folded zones of the non-cubic cases. For the the bulk transport calculation we used a 32×32×32 mesh.
